# Feasibility of an educational program for public health nurses to promote local healthcare planning: protocol for a pilot randomized controlled trial

**DOI:** 10.1186/s40814-022-01054-8

**Published:** 2022-04-27

**Authors:** Kyoko Yoshioka-Maeda, Takafumi Katayama, Misa Shiomi, Noriko Hosoya, Hitoshi Fujii, Tatsushi Mayama

**Affiliations:** 1 Department of Community Health Nursing, Division of Health Sciences and Nursing, Graduate School of Medicine, The University of Tokyo, 7-3-1 Hongo, Bunkyo-ku, Tokyo, 113-0033 Japan; 2 Department of Statistic and Computer Science, College of Nursing Art and Science, University of Hyogo, Hyogo, Japan; 3 Department of Human Health Sciences, Graduate School of Medicine, Kyoto University, Kyoto, Japan; 4 Department of Nursing, Faculty of Healthcare Sciences, Chiba Prefectural University of Health Sciences, Chiba, Japan; 5 Department of Medical Statistics, School of Nursing, Mejiro University, Saitama, Japan; 6 Faculty of Policy Studies, Doshisya University, Kyoto, Japan

**Keywords:** Competency, Continuing education, Local healthcare planning, Public health nursing, Randomized controlled trial, Web-based education

## Abstract

**Background:**

Promoting of local healthcare planning is crucial for assisting public health nurses in improving community health inequities. However, there is no effective educational program for developing relevant skills and knowledge among these nurses. Therefore, this study aims to assess the feasibility of a newly developed web-based self-learning program to promote the involvement of public health nurses in the local healthcare planning process.

**Methods:**

A pilot randomized control trial randomly allocated eligible public health nurses to intervention and control wait-list groups [1:1]. The former will be exposed to six web-based learning modules from July to October 2021. After collecting post-test data, the wait-list group will be exposed to the same modules to ensure learning equity. The primary outcome will be evaluated by implementing a validated and standardized scale designed to measure public health policy competencies at the baseline and post-intervention, while secondary outcome will be measured on an action scale to demonstrate the necessity of healthcare activities. The third outcome will be the knowledge and skills related to local healthcare planning by public health nurses. The participants will provide feedback through free descriptions on the trial feasibility and a web-based self-learning program to identify improvement points for continual refinement.

**Discussion:**

The results will provide suggestions in preparation for a future definitive randomized controlled trial. This will provide preliminary data for an intervention aimed at improving relevant competencies among public health nurses who are tasked with resolving health inequities in their respective communities through local health planning.

**Trial registration:**

The protocol for this study was registered with the University Hospital Medical Information Network Clinical Trials Registry and approved by the International Committee of Medical Journal Editors (No. UMIN000043628, March 23, 2021).

**Supplementary Information:**

The online version contains supplementary material available at 10.1186/s40814-022-01054-8.

## Background

Health inequities and community health are important areas of focus across the world. Public health policies should target health gaps to resolve current and future community health needs [1]. Interventions should focus on the social determinants of health which are the root causes of health inequities, to reduce vulnerabilities and address the unequal consequences of illness [2]. In particular, local governments must develop public health policies and effective inter-sectoral programs [3]. Additionally, public health nurses (PHNs) are responsible for improving a variety of health inequities, including the lack of limited access to healthcare services [4], as their unique professional roles provide a way to directly identify and address specific community health needs [5]. Local healthcare planning is a crucial strategy that PHNs can implement to reduce health inequities and bridge the gaps between community health needs [6]. In Japan, local healthcare plans include securing hospital beds, health and welfare services, and resources in each region to improve community health needs and equitable distribution of care [7].

PHNs may face challenges when developing competencies relevant to local healthcare planning [8, 9]. A systematic review demonstrated their necessary competencies, such as reflecting the narratives of community residents identified through providing individual care [9], determining the necessary programs and building consensus among those concerned [10, 11], and understanding policy and securing budget [12, 13]. To enhance these competencies and self-confidence among PHNs, continued education should be implemented [14, 15]. Web-based learning programs provide more advantages than traditional face-to-face group sessions and are often more flexible, user-friendly, convenient, and time-saving [16, 17]. However, there have been no randomized controlled trials (RCT) in this field [18, 19], and little is currently known about the effectiveness of web-based learning for PHNs [13, 17].

In Japan, approximately 60% of PHNs work for prefectural or municipal governments [20]. The national practice guidelines for PHNs published in 2013 increased their expected involvement in local healthcare planning [7]. Undergraduate curricula began to include these skills in 2007 [21], and the national PHN examination added related requirements in 2017 [22]. However, approximately 50% receive continued education relevant to local healthcare planning [23, 24]. Previous studies have not investigated whether PHNs who focused on self-directed learning improved self-confidence, competency, and readiness [25, 26].

Japan is facing a falling birth rate and an aging population. Consequently, local governments are promoting the reduction of public service positions despite the necessity of needs-oriented local healthcare plans [27]. The COVID-19 pandemic has also accelerated the growing shortage of PHNs [28], which emphasizes the need for effective and efficient educational programs targeted at improving competencies related to local healthcare planning. Thus, a web-based learning program would help PHNs understand theoretical concepts and practical strategies that are relevant to the healthcare planning process whenever they want without the need to contact other participants. Subsequently, this study will verify the effects/feasibility of a user-friendly web-based self-learning program designed to improve competencies for PHNs who are involved in the local healthcare planning process. Further, the study will verify the program outcomes and data collection methods using relevant scales and examine the preliminary effects of the web-based self-learning program, thus providing relevant data for calculating the sample size needed to conduct a future definitive RCT.

## Methods

### Trial design and settings

A pilot prospective RCT design will be implemented among single-blind participants and parallel groups. To ensure equal learning opportunities for both groups, PHNs who will answer a pre-test survey and register for this study will be randomly allocated to the intervention or wait-listed control groups [1:1]; as such, a double-blind design was not possible. All PHNs will be exposed to the same self-learning program; the intervention group will participate in the program first, and then, the wait-listed control group will be offered the same program. All participants will answer a post-test survey.

### Ethical considerations

The Institutional Review Board (IRB) at the organization affiliated with the primary researcher approved the protocol for this study on February 17, 2021 (ID: NIPH-IBRA#12313). The IRB of the third researcher also approved the study protocol on March 16, 2021 (ID: C1516).

This pilot study was registered with the University Hospital Medical Information Network Clinical Trials Registry (UMIN-CTR) following the International Committee of Medical Journal Editors (No. UMIN000043628, March 23, 2021). Explanations of the study’s aims and procedures will be provided to the participants through the study website. All eligible PHNs will provide written web-based informed consent prior to the study registration. Only those who will meet the inclusion criteria and agreed to participate will be included in the study. To ensure privacy, no personal information, including place of employment, will be collected.

### Participant eligibility criteria

Eligible participants will include PHNs who are full-time employees in Japan and have experience in local healthcare planning. Exclusion criteria will include those who do not have Internet access, are employed part-time, and have no opportunity to be involved in the local healthcare planning process.

### Sample size

The sample size has not yet been calculated using a power analysis due to feasibility and a limited research budget [29]. However, a rationale for numbers in the pilot trial should be included for pilot studies [30]. Based on our previous RCT, we estimate the participation rate would be approximately 2.4% [17]. In this pilot study, we will focus on a prefecture in the Kanto region in Japan, which has approximately 600 PHNs and 1,930,000 residents. Therefore, we will estimate that 14 PHNs were necessary for this study. Owing to ethical considerations, we do not over-recruit the participants. Further, we expect the participant pool to be limited due to the COVID-19 pandemic, which has resulted in a general shortage of PHNs because most will be involved in frontline activities [28].

### Sampling, informed consent, and randomization

The research team will send 36 invitations to participate (i.e., one to the prefectural government and 35 to the municipal governments) to public health nursing directors in May 2021 and a reminder letter in early June 2021. These directors will be asked to share information about this study with their PHNs. Those who will express interest in participation will be given access to our study website, which will contain a study explanation sheet and written web-based informed consent forms for completion. Only those who will agree to these conditions and will meet the eligibility criteria (as determined by the researchers) will be allowed to participate. The registration period will be from May until the end of June 2021. To ensure privacy, the participants will be registered using pseudonyms and their e-mail addresses.

**Fig. 1:**
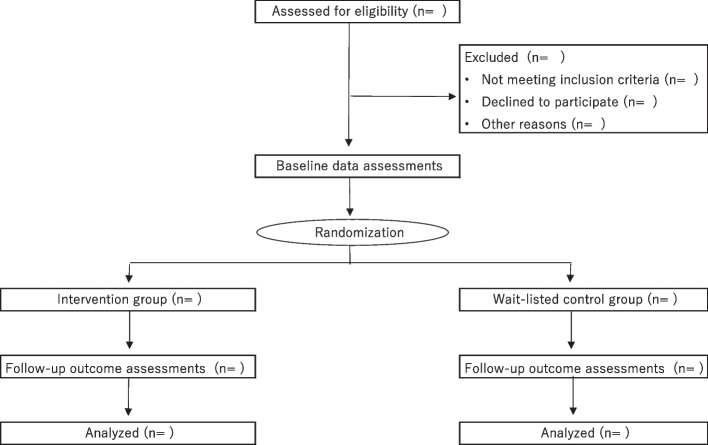
Consolidated Standards of Reporting Trial (CONSORT) flowchart

Figure 1 illustrates a flowchart for the Consolidated Standards of Reporting Trial (CONSORT) design, which will be used throughout the study process. Randomization will be conducted by a researcher specializing in statistics and the co-author via an equal computer-based randomization table. A permuted-block method will be adopted to ensure that the participants will be equally divided between the intervention and control groups. In total, six blocks will be used with four sizes. We will send an e-mail to all participants informing them of their assigned group.

### Minimizing bias

All data will be anonymized, and we will not collect any personal information or the names of the local governments where the participants are employed. The data collection will be conducted through the study website, where the participants will enter the relevant information and will complete the required elements (e.g., demographic data and the outcome scales); this information will be evaluated as baseline and post-test data. The intervention group will be asked to keep the contents of the web-based self-learning program confidential so that the control group will not be exposed to any such information. The risks of contaminating the intervention group will be explained on the study website, and the intervention group will receive a URL password-protected (passwords sent via e-mail) address to view the learning modules.

### Description of the intervention: overview of the web-based self-learning program

We designed an outline of the web-based self-learning program, which included six modules for acquiring knowledge and skills related to local healthcare planning based on adult learning theory [31], a review of the relevant Japanese literature [13], and several specific studies [5, 7, 16, 17, 23, 24, 32]. In the pre-test, five PHNs with previous involvement in local healthcare planning will be asked to confirm the modules and content. We then added explanations for each module and confirmed that the newly developed web-based self-learning program will include knowledge and skills that PHNs could use to promote local healthcare planning effortlessly and effectively. Each module takes approximately 10–20 min to complete.

Table 1 presents the themes and objectives for each module as follows: (1) create a concrete vision of the ideal community, (2) bridge the daily practices of PHNs’ with local healthcare planning, (3) basic knowledge necessary for local healthcare planning, (4) understand the steps of the healthcare planning process, (5) hone coordinate skills with related parties, and (6) accomplish goal setting and project evaluation. The participants can use this worksheet to identify and summarize the objectives for local healthcare planning, community health needs, related government plans, legal issues, relevant staff and key persons in the community, and an evaluation index. The intervention will be conducted from July to August 2021. Once the post-test data will be collected in September 2021, the control group will participate in the program from October to November 2021.

**Table 1:** Themes and objectives of each web-based self-learning module

	Module themes	Objectives
1	Create a concrete vision of the ideal community	Understand the viewpoints of creating a concrete vision of the ideal community
2	Bridge the daily practices of PHNs' with local healthcare planning	Envision the implementation of the Plan-Do-Check-Action cycle, from the evaluation of daily PHN practices to clarifying improvements and procedures Increase the motivation to bridge the daily practices of PHNs with the PDCA cycle
3	Basic knowledge necessary for local healthcare planning	Sort out the policies, programs, and projects involved in the plan you are helping to develop Understand the relationship between the plan involving PHNs and various other plans
4	Understand the steps of the healthcare planning process	Understand the overall schedule and development process of planning Understand the nature of goal setting for each program and project
5	Hone coordination skills with related parties	Understand how to set up the committees/meetings necessary for planning Understand how to collaborate with related persons and residents in order to utilize the plan in daily PHN practices
6	Accomplish goal setting and project evaluation	Understand the list of target values related to national/prefectural governments Establish project objectives that are consistent with the target values of national/prefectural governments

### Outcomes and data collection

Using the study website, the eligible participants will provide demographic data and will complete the outcome scales at the baseline and post-intervention. More specifically, demographic data will include gender, age, years of PHN experience, job title, educational background, affiliation, previous experience with local healthcare planning education at the undergraduate level and on-the-job training after graduation, previous experience with local healthcare planning, whether they worked with colleagues who played active roles in healthcare planning, and information on promoting the daily practices of PHNs. To decrease the number of dropouts, we will send e-mails to all participants with information about the study progress and will remind them to answer the post-test questions. At the baseline and post-intervention, the participants will address the following outcomes.

#### Competencies related to involvement in health policy

The primary outcome will be measured using a self-reported 16-item scale targeting PHN competencies in public health policy in the context of Japanese local governments [33]. The scale was confirmed as reliable and valid for use in Japan and is only available in Japanese. The first eight items measure community partnership among PHNs when developing health policy, while the remaining items evaluate competencies related to the community nursing diagnosis cycle. All items will be measured using a 4-point Likert scale, ranging from 0 to 3: 0 (unable to do), 1 (partially unable to do), 2 (partially able to do), and 3 (able to do).

#### The necessity of healthcare activities

The secondary outcome will be measured using a self-reported 19-item scale designed to reveal information about the necessity of healthcare activities that are based on evidence and the promotion of decision-making with superiors as a way to improve planning [34]. This scale was confirmed as reliable and valid for use in Japan and is only available in Japanese. The 19 items listed in the scale are organized within four dimensions as follows: (1) three items measured the existence of health needs, (2) five items measure the necessity of addressing health needs based on evidence, (3) five items measure actual conditions that require solutions, and (4) six items measure priorities for resolution. All items will be measured using a 6-point Likert scale: 0 (never applicable), 1 (20% applicable), 2 (40% applicable), 3 (60% applicable), 4 (80% applicable), and 5 (100% applicable).

#### Knowledge, skills, and perspectives of PHNs involved in local healthcare planning

The third outcome will be measured based on 29 questions targeting the knowledge, skills, and perspectives of PHNs involved in local healthcare planning. All items will be measured using a 4-point Likert scale: 1 (never), 2 (not much), 3 (a little), and 4 (a lot).

### Preliminary effects

We will expect the trial to improve relevant competencies among PHNs measured as three outcomes.

### Assessment of the trial feasibility

We will assess the feasibility of this study based on the recruitment rate, dropout rate, and data completion rate. We will set the corresponding criteria to assess the success of feasibility; the recruitment rate will be 2.4%, the dropout rate, and the data completion rate will be 50% based on the previous pilot study [12]. We also will ask the participants for their feedback on the feasibility of the trial and web-based learning self-learning program, clarifying areas that need improvement and/or continual refinement as open-ended survey questions.

### Data management

As there will be no harmful interventions, we will not develop a data monitoring committee. Researcher TK will be responsible was not established. Researcher TK will be responsible for managing all the data obtained through the study website and will alert the research team about any recruitment or data collection problems. Researcher TK will provide all data to the research team via Microsoft Excel (password locked), and the research team will conduct a quality assurance procedure.

### Statistical analysis

We will conduct descriptive statistics (e.g., recruitment rate, dropout rate, and data completion rate) to assess the feasibility of this study and analyze intergroup baseline differences in the data and primary outcomes using the Mann-Whitney *U*-test and the chi-square/Fisher exact test. We will assess whether previous experience in local healthcare planning and other factors affected the study outcomes based on the results of the Mann-Whitney *U*-test and the chi-square/Fisher exact test. Analyses will be performed according to the intention-to-treat principle. We will use IBM SPSS for Windows (version 25; IBM Corp, Armonk, NY, USA) for the analysis and *p*-values < .05 indicating statistically significant intergroup differences. This study is expected to have a power ≥.80 and α ≤.05, assuming a medium effect size (*d* = .30) [35].

### A content analysis

We will conduct a content analysis using all participant feedback provided through the free descriptions mentioned earlier, specifically regarding their feasibility and the web-based self-learning program itself. This will be done by entering relevant information into a Microsoft Excel worksheet. We will code all data and sort the results based on their commonalities. The positive comments will help assess the feasibility of this study, and the negative comments will contribute to improving the program.

## Discussion

This pilot RCT will aim to confirm the feasibility and preliminary effects of a web-based self-learning program designed to increase the involvement of PHNs in local healthcare planning by enhancing relevant competencies. We will also examine the eligibility of the implemented outcome scales and data collection procedures in preparation for a future definitive RCT. The strengths of the novel self-learning program are as follows: (1) user-friendly web-based educational program, (2) eligible participants will be selected based on clear criteria, (3) all participants will be given equal opportunities to learn about local healthcare planning through the program (i.e., after we collect data from the intervention group), and (4) primary outcomes will be measured using validated standardized scales. This study also has the strength that PHNs will understand theoretical concepts and practical strategies relevant to healthcare planning without contact with other participants due to the COVID-19 pandemic.

We developed the abovementioned program based on several components, including adult learning theory [31], a nationwide survey [25], and previous related studies [5, 16, 17, 21, 23, 25]. Through participation in web-based self-learning modules, the participants can acquire the competencies of local healthcare planning and bridge the gaps between the community health needs that PHNs must address in daily practice and the context of newly developed local healthcare plans. The development of local healthcare planning can improve health inequities by bridging the gaps in community health needs [6]. In this regard, PHNs are responsible for enhancing health equity and developing needs-oriented health policy through their practices [4, 5]. This pilot feasibility study will also adopt validated outcome scales, which will be used to gather the preliminary data needed to calculate an appropriate sample size for a future large-scale RCT. Owing to the constraints of the COVID-19 pandemic, we will also ensure that participants are socially distanced to prevent infection. More specifically, our web-based learning modules do not require participants to make direct contact with one another and will also eliminate travel time.

As mentioned, participants in the control group can access the same learning program in the post-intervention context, providing them with critical education while minimizing the number of dropouts and ensuring educational equality. Further, we will assess whether previous experience with local healthcare planning impacts the study outcomes. These results will be used to revise different aspects of the program and the inclusion criteria for a future definitive RCT.

We have also identified some potential limitations. First, the pilot study only included a small sample from one prefecture in Japan. In this regard, the participants will be likely to have strong interests in the nature of the study program, thus increasing their motivation to engage in continued education. This may result in selection bias and influence the distribution of data. However, this study will focus on the feasibility of the program, not its effectiveness. Regardless, these issues should be carefully considered when interpreting the data.

Second, we will protect the privacy of all participants by anonymizing the data and did not collect the names of the local governments where they are employed. As such, we cannot rule out the possibility that eligible participants from the same local governments will be allocated to different study groups. In this regard, we cannot entirely prevent contamination or instances in which the program content is leaked from the intervention group to the wait-listed control group. We will explain this issue to all participants and repeatedly ask the intervention group to keep the program contents confidential.

Third, we could not control some confounding factors, such as the provenance area of participants and rural versus urban areas and the lack of follow-up assessment in this study. Fourth, only five PHNs confirmed the web-based self-learning modules due to the COVID-19 pandemic. Fifth, the reliability and validity of the Japanese version of Fisher’s Self-Directed Learning Readiness Scale has not been confirmed. Thus, we could not use this scale in this study.

Despite these limitations, this pilot RCT will assess the feasibility of a newly developed web-based self-learning program designed to increase the involvement of PHNs in local healthcare planning while also providing the preliminary data needed for a future definitive RCT in Japan. Therefore, we will confirm whether the web-based, self-learning system is considered user-friendly and whether participants exhibit improved competencies in the local healthcare planning context following the intervention.

## Conclusions

This pilot RCT will determine the feasibility of a novel web-based, self-learning program for PHNs in local healthcare planning. We will also confirm the appropriateness of the enrollment process and data collection procedures. Further, the pilot study results will provide preliminary data and essential feedback for use in a future main RCT. Here, the challenge is to provide new evidence for improving competencies among PHNs who are tasked with resolving health inequities in their respective communities via local healthcare planning.

### Trial status

Trial registration: No. UMIN000043628, registered on March 23, 2021. Enrolling and date recruitment began: April 22, 2021. Date recruitment completed: June 30, 2021. Latest protocol version approved February 17, 2021, National Institute of Public Health and 2021 (NIPH-IRBA#12313), and approved March 16, 2021, Graduate School of Medicine, Kyoto University (C1516).

## Supplementary Information

**Additional file 1: Appendix.** Items of knowledge, skills, and perspectives of PHNs involved in local healthcare planning (The third outcome).

## Data Availability

Not applicable.

## Abbreviations

IRB: Institutional Review Board
PHNs: Public health nurses
RCT: Randomized controlled trial
