# Correction: Feasibility of an educational program for public health nurses to promote local healthcare planning: protocol for a pilot randomized controlled trial

**DOI:** 10.1186/s40814-023-01343-w

**Published:** 2023-06-22

**Authors:** Kyoko Yoshioka‑Maeda, Takafumi Katayama, Misa Shiomi, Noriko Hosoya, Hitoshi Fujii, Tatsushi Mayama

**Affiliations:** 1 Department of Community Health Nursing, Division of Health Sciences and Nursing, Graduate School of Medicine, The University of Tokyo, 7‑3‑1 Hongo, Bunkyo‑ku, Tokyo, 113‑0033 Japan; 2 Department of Statistic and Computer Science, College of Nursing Art and Science, University of Hyogo, Hyogo, Japan; 3 Department of Human Health Sciences, Graduate School of Medicine, Kyoto University, Kyoto, Japan; 4 Department of Nursing, Faculty of Healthcare Sciences, Chiba Prefectural University of Health Sciences, Chiba, Japan; 5 Department of Medical Statistics, School of Nursing, Mejiro University, Saitama, Japan; 6 Faculty of Policy Studies, Doshisha University, Kyoto, Japan

**Correction: Pilot Feasibility Stud 8, 92 (2022)**

**https://doi.org/10.1186/s40814-022-01054-8**

Following publication of the original article [1], an error was identified under Description of the intervention: overview of the web‑based self‑learning program.

The sentence currently reads:

We designed an outline of the web-based self-learning program, which included six modules for acquiring knowledge and skills related to local healthcare planning based on adult learning theory [31], a review of the relevant Japanese literature [14],

The sentence should read:

We designed an outline of the web-based self-learning program, which included six modules for acquiring knowledge and skills related to local healthcare planning based on adult learning theory [31], a review of the relevant Japanese literature [13],

The original article [1] has been corrected.
