# Supplementary material for: Feasibility of an educational program for public health nurses to promote local healthcare planning: protocol for a pilot randomized controlled trial
Source: Pilot Feasibility Stud. 2022 Apr 27;8:92. doi: 10.1186/s40814-022-01054-8 (PMC9043510; doi:10.1186/s40814-022-01054-8)
Supplement: Supplementary file 1 — Additional file 1: Appendix. Items of knowledge, skills, and perspectives of PHNs involved in local healthcare planning (The third outcome). [file 40814_2022_1054_MOESM1_ESM.docx]

Appendix: Items of knowledge, skills, and perspectives of PHNs involved in local healthcare planning (The third outcome)

| Web 1 | 1 | Organizing residents’ opinions into demands and needs. |
| --- | --- | --- |
|  | 2 | Understanding that the worst and the best are not always two sides of the same coin. |
|  | 3 | Understanding that too much waste will lead to insufficient community outreach. |
|  | 4 | Understanding that numerical indicators are only one of the points of passage. |
| Web 2 | 5 | Willingness to use reflections on daily practices of PHNs in local healthcare planning. |
|  | 6 | Understanding the significance of using local healthcare planning as an opportunity to evaluate daily practices of PHNs and implement the PDCA cycle. |
|  | 7 | Understanding how to evaluate daily practices of PHNs. |
|  | 8 | Understanding how to find improvement measures from evaluation and apply them to local healthcare planning. |
|  | 9 | Be able to plan specific measures to find improvements from the evaluation. |
| Web 3 | 10 | Understanding the difference and usage of policies, programs, and projects. |
|  | 11 | Be able to recognize which work you are responsible for in items of the policies, programs, or projects. |
|  | 12 | Understanding the relationship between upper-level and lower-level local healthcare planning. |
|  | 13 | Be able to list higher-level or lower-level plans that are related to the plan you are involved in. |
|  | 14 | Understanding the interconnectedness of various related plans within the field of your local government. |
|  | 15 | Be able to list specific plans in your local government area, related fields, and other fields that are relevant to the plan you are involved in. |
| Web 4 | 16 | Understanding the overall schedule for developing the local healthcare plan. |
|  | 17 | Knowing which tasks in the planning process are to be completed and by when. |
|  | 18 | Understanding the rationale for considering the cancellation of a program or project. |
|  | 19 | Identifying health issues that need to be addressed based on the evaluation of the previous plan. |
|  | 20 | Understanding that outcome measures are often used for targets. |
|  | 21 | Understanding how to set targets. |
| Web 5 | 22 | Understanding the meetings that need to be established for local healthcare planning. |
|  | 23 | Understanding the advance preparations and procedures required to set up a meeting. |
|  | 24 | Applying the local healthcare plan to the daily practices, select stakeholders, and residents who will participate. |
|  | 25 | Understanding how to share objectives with internal and external stakeholders and residents, and the methods necessary for coordination and consensus building. |
|  | 26 | Understanding the importance of sharing objectives and building cooperative systems within your own organizations. |
| Web 6 | 27 | Be able to clarify local health issues using health checkup data, data from KDB (National Health Insurance Database System). |
|  | 28 | Be able to set numerical indicators for solving priority health issues. |
|  | 29 | Understanding the contents of the national (Health Japan 21) and prefectural health promotion plans. |
